# A Comprehensive Review of Organ-on-a-Chip Technology and Its Applications

**DOI:** 10.3390/bios14050225

**Published:** 2024-05-01

**Authors:** Negar Farhang Doost, Soumya K. Srivastava

**Affiliations:** Department of Chemical and Biomedical Engineering, West Virginia University, Morgantown, WV 26506, USA; nf00011@mix.wvu.edu

**Keywords:** organ-on-a-chip, disease models, multi-organs-on-a-chip, human-on-a-chip

## Abstract

Organ-on-a-chip (OOC) is an emerging technology that simulates an artificial organ within a microfluidic cell culture chip. Current cell biology research focuses on in vitro cell cultures due to various limitations of in vivo testing. Unfortunately, in-vitro cell culturing fails to provide an accurate microenvironment, and in vivo cell culturing is expensive and has historically been a source of ethical controversy. OOC aims to overcome these shortcomings and provide the best of both in vivo and in vitro cell culture research. The critical component of the OOC design is utilizing microfluidics to ensure a stable concentration gradient, dynamic mechanical stress modeling, and accurate reconstruction of a cellular microenvironment. OOC also has the advantage of complete observation and control of the system, which is impossible to recreate in in-vivo research. Multiple throughputs, channels, membranes, and chambers are constructed in a polydimethylsiloxane (PDMS) array to simulate various organs on a chip. Various experiments can be performed utilizing OOC technology, including drug delivery research and toxicology. Current technological expansions involve multiple organ microenvironments on a single chip, allowing for studying inter-tissue interactions. Other developments in the OOC technology include finding a more suitable material as a replacement for PDMS and minimizing artefactual error and non-translatable differences.

## 1. Introduction

Microfluidics is the scientific technology that manipulates minute fluid volumes, typically between 10^−9^ and 10^−18^ L [1]. This is made possible by utilizing small channels; these channels’ dimensions range from ten to a couple of hundred micrometers. Microchannels of this size have a large relative surface area and, consequentially, high mass transfer. Other inherent benefits of microfluidics include faster mixing rates, increased control of volumes, and other properties such as low reagent use and prompt response [2,3]. Microfluidics has been impacting biological research for some time, having been integrated into processes for sample preparation and different cell culture applications [4].

Organ-on-a-chip technology utilizes microfluidics to simulate in vivo research while maintaining the controlled parameters in vitro research provides. Standard cell biology research relies on in vitro research, that is, research performed in a test tube, culture dish, or elsewhere outside the living organism. OOC technology aims to overcome these challenges of in vivo and in vitro research [2]. 

OOC technology utilizes low sample volumes, ranging anywhere from microliters to femtoliters. Such small volumes of liquid drastically reduce its Reynolds number. The Reynolds number is a dimensionless quality used to predict fluid flow patterns under various flow situations. A low Reynolds number indicates laminar flow, whereas a high Reynolds number indicates turbulent flow.

Microfluidics has significant advantages over working on a macro scale, including increased efficiency, lower cost, and increased control of volume and other experimental parameters [5]. OOC technology utilizes microfluidics to provide a state-of-the-art platform for biological research that combines the advantages of in vivo and in vitro research [6].

## 2. Methods

Microfluidics was first implemented in gas chromatography systems and printing machines to perform fast, precise analysis on small volumes [1]. Later, microelectronic lithography was adapted to construct devices from biocompatible materials for medical purposes. Since then, increasing interaction between microelectromechanical systems (MEMSs) and microfluidics has led to a novel fluidic device fabrication method that uses photo-sensible polymers to produce molds using UV light, which is then used to cast a pattern onto a biocompatible polymer [7]. The growing utilization of microfluidics in technology has led to the development of many other fabrication processes using novel materials. Replica molding, injection molding, and microprinting are a few techniques used to create microfluidic platforms [8,9,10]. Polydimethylsiloxane remains the standard material used in fabrication due to its transparency, flexibility, permeability, and biocompatibility. 

Recently, three-dimensional (3D) bioprinting technology has revolutionized the printing of microfluidic devices. It can integrate channels and connections in organ-on-a-chip models [11]. Three-dimensional printing technology reduces the fabrication process of microfluidic devices to a shorter time, making it less cumbersome. It also has considerable advantages over traditional fabrication processes. These include the ability to incorporate multiple biomaterials, including living cells, controlled porosity of tissue scaffolding, and record accuracy. This technique can be divided into nozzle-based and optical-based methods [11]. Nozzle-based methods involve the use of a nozzle that applies the ink onto a substrate, typically with adequate pressure to ensure ink transfer, while optical-based 3D printing can mean either direct printing or transferring the ink onto a receiving substrate through ejection or by utilizing light–material interactions to crosslink the ink [11]. Three-dimensional printing technology utilizes various starting materials, including photocurable resin, photopolymers, thermoplastic polymers, and hydrogels [12]. PDMS remains the preferred choice in microfluidics, but other potential materials are continuously developing for various biological research applications. Some of these materials are tabulated below in Table 1. 

To choose the best material according to the organ-on-a-chip application, one should consider surface properties such as surface roughness and wettability, which affect cell adhesion and migration, and mechanical properties such as stiffness, which influence the later stages of cell growth [38]. Analyses such as tensile strength, compressive stress, and wettability are commonly conducted to assess the suitability of polymer properties for the intended application [38]. For the fabrication of porous-compartment-separation membranes, synthetic polymers like polydimethylsiloxane (PDMS), polycarbonate (PC), polyethylene terephthalate (PET), aliphatic polyesters, and polyurethanes, among others, have been utilized due to their ability to adjust porosity, surface roughness, and mechanical properties easily. In contrast, natural polymers such as collagen, gelatin, or polysaccharides have gained recent attention for their enhanced biocompatibility and replication of native tissues due to facilitation in the creation of channel interconnections, enabling the perfusion of oxygen and nutrients to mimic the natural behaviors of cell differentiation, spreading, and adhesion [38].

### 2.1. The History of Organ-on-a-Chip

Huh et al. [39] published the first “lung-on-a-chip” article in 2010, introducing the idea of “organ-on-a-chip” as microfluidic in vitro cell culture systems that mimic the physiology of the basic functional units of an organ. A public–private partnership assigned Ingber’s lab to develop ten human organs-on-a-chip two years later. The business has created liver and intestine models in addition to a lung-on-a-chip, according to Hamilton, and is currently working on the next generation, which will include brain, kidney, and skin chips [40]. Organ-on-a-chip technology is growing in popularity for biological research due to the high expectations for reproducing essential elements of the human physiological environment and the ability to replace animal models. Organ-on-a-chip technology could speed up the time it takes for therapeutic compounds to enter clinical trials by reducing the reliance on animal testing, which is now the primary method used to evaluate the kinetics, efficacy, and safety of medication candidates. Also, studying pharmacokinetics in particular persons, i.e., personalized, is possible by cultivating human cells on chips [41]. 

### 2.2. Surface Treatment of OOC Devices

To ensure biocompatibility or improve cell adherence, microfluidic device surfaces that are in contact with cells must be treated [42]. Treatment with pluronic acid is often used in 3D spheroid or organoid cultures to passivate the chip surface and avoid unwanted spheroid or organoid breakup through possible cell attachment [42]. This is critical because the loss of 3D tissue architecture may result in a loss of physiological organ function [42]. On the other hand, protein and extracellular matrix (ECM) coatings may be employed to increase cell adhesion to the chip substrate [42]. These coatings are required to produce connected, confluent monolayers of cells, which mimic the intestinal epithelium in the gut or the endothelium in the blood–brain barrier (BBB) OOC [43,44,45]. Tissue-specific and disease-specific matrices may produce higher-fidelity pathophysiological models, such as generating crypt-like formations in gut-on-a-chip systems [46]. They may be basic biomatrices formed from fibrin or collagen or more complicated biomatrices like Matrigel. The liquid form of these biomatrices may be combined with cells before being fed into the OOC device and allowed to polymerize into a gel. Biomatrices may act as a helpful framework for cells to remodel into a 3D structure since certain organs, like the liver and skin, need a 3D microenvironment to achieve physiological functioning. Additionally, biomatrices could be necessary for the upkeep or differentiation of certain cell types. For instance, it has been shown that Matrigel encourages neural stem cells to maintain and differentiate their neurons, while myogenic differentiation was increased when myoblasts were cultured on a suspended overhanging fibrin-based gel [47,48].

Another method, micropatterning, is a powerful tool to control the spatial organization of cells and tissues to mimic the in vivo microenvironment closely. A recent study by Fang et al. demonstrated the potential of micropatterning technology to study tubulogenesis in epithelial organs and the differentiation of mesenchymal stem cells [49]. Moreover, Nguyen et al. quantified the electrical interaction between cardiomyocytes and other cells in micropatterned cell pairs [50]. These two studies showed the diverse application of micropatterning in OOC systems. 

## 3. Supporting Life inside the Device

### 3.1. Cell Culture Media Selection

It is also possible to use culture media designed for traditional cultures for a single-OOC platform involving just one kind of cell. When developing single- or multi-OOC platforms containing numerous cell types, each with a different nutritional demand, a new level of complexity for media selection is introduced [42]. Each unique cell population must retain its viability and functional phenotype in the optimum co-culture medium [42]. This condition must be met before assessing the appropriateness of the media for supporting downstream analysis without unwanted interference [42]. Many research groups have employed combinations of the original culture media used for each distinct cell type to optimize a suitable co-culture medium for OOC applications, with mostly positive results [42]. These include multicellular OOC models simulating adipose tissue and multi-organ OOC models simulating liver–kidney and liver–adipose–skin–lung interactions [42]. However, optimizing an excellent co-culture medium becomes increasingly difficult when more diverse cell types are co-cultured. Cells can be divided into distinct compartments through permeable membranes without needing a common co-culture medium. This allows for the delivery of cell-specific media to each compartment while allowing paracrine interactions between the compartments, as demonstrated by OOC devices that mimic the skin [51] and liver [52]. Since sera are a significant source of variance and may affect tests, a serum-free medium should be considered when practical. Starting with the medium that is most often used for non-OOC work with the same cells, such as minimum essential medium (MEM)-based medium for epithelial cell cultures or endothelial cell growth medium (ECGM) for endothelial cells, media should be improved in research with the OOC [53].

### 3.2. Cell Microenvironment Control

In cellular environments, interactions with soluble factors, the extracellular matrix (ECM), and neighboring cells occur within a 3D milieu characterized by specific physical and chemical properties, collectively referred to as the cell microenvironment. Precision control of this microenvironment is essential for proper cellular function in OOC systems, which offer superior control to conventional bulk cell culture systems. The microscale resolution of OOC allows for the precise definition of culture chamber geometry and associated physical and chemical phenomena of fluids. Microfluidic control of environmental factors is often coupled with fluid shear stress and mass transport regime functions of flow velocity [42]. 

Over-the-chamber systems have been widely used to manipulate various environmental factors (including shear stress, autocrine/paracrine soluble factors, and cell–cell and cell–ECM interactions) at physiologically relevant lengths and timescales to elucidate their effects on cell phenotypes and functions. This concept is best exemplified in the context of stem cell and tumor microenvironments, as seen by the large number of reviews covering these areas [54]. Over-the-chamber systems provide a more precise method to study the effects of paracrine and autocrine signaling than conventional techniques, such as conditioned medium or changing cell density, because soluble biochemical factors can be controlled in over-the-chamber systems by manipulating fluid mass transport properties at a microscale resolution. A switch may be made between diffusion-dominated transport, in which secreted factors stay close to cells for receptor binding, and convection-dominated transport, in which secreted factors are carried away from cells [55]. Over-the-chamber devices are useful for stimulating cells in various physical environments. The shear stresses produced by the body’s fluids, such as blood and interstitial fluids, are often mimicked with over-the-chamber systems since fluid flow is an intrinsic part of many systems. One may create multiplexed channels with variable geometries to simultaneously apply shear stress across a range of magnitudes to investigate the impact of shear stress on cell proliferation, differentiation, and function. In contrast, fluid shear promotes microvascular development and function in various organotypic tissues. Mechanical tensile or compressive pressures may be applied to cells using over-the-chamber devices to simulate the stretching of the airway during breathing, the movements of the digestive system during peristalsis, or the contractility of cardiac tissues. To accomplish this, over-the-chamber devices often feature thin, flexible PDMS membranes stretched cyclically by vacuum or pressure pneumatic actuators, allowing cells to be grown in a fluidic environment. These devices have included in situ microfabricated microelectrode arrays as part of the device or electrodes placed into the system to better mimic the electrophysiological activities of neuronal and cardiac tissues via electrical stimulation [55,56].

Three-dimensional culture, multi-cell-type co-culture, and tri-culture systems aided researchers in creating various in vitro complex models to mimic in vivo environments and provide valuable information. In the context of cell cultures, adding a third dimension requires knowledge about the design of the scaffolds to support the cell organization or the utilization of bioreactors to control the nutrient/waste exchange [57]. A 3D cell culture is widely used in drug screening and tissue engineering [57].

Huh et al. studied the importance of 3D cell cultures, which enhance cell differentiation and tissue organization compared to 2D models [58]. Other groups underscored the significance of the multi-cell-type co-culture in producing multi-organoid-on-a-chip and designing human models with better physiological mimicry [59,60]. The study by Wagner et al. showcased the interaction of natural and drug-induced liver–testis models derived from primary adult testicular cells and liver spheroids consisting of cultured HepaRG cells and hepatic stellate cells to demonstrate the implications of multi-cell-type co-cultures [61]. 

### 3.3. Post-Experiment Analysis

Intermittent simultaneous measurements of different cell parameters are ideal for profiling OOC homeostasis and responses to externally applied chemical or physical stressors. Some standard techniques include multiplexed (bead-based) protein-binding/DNA-binding assays or offline high-performance liquid chromatography/mass spectrometry (HPLC/MS), which enable the simultaneous measurement of various chemicals, many being responsive biomarkers [62]. Both offline methods may be carried out using microliter sample quantities, consistent with the typical flow rates used in one-pass perfusion, or by periodically removing recirculating medium circuits. For some biomarkers (such as albumin, lactate dehydrogenase, alanine aminotransferase, aspartate aminotransferase, and urea for liver systems), different commercial assay kits can be used as an alternative; however, any potential interference from medium components (particularly serum) must be carefully considered. The number of measurements that can be performed with these kits in extremely low-volume systems is somewhat restricted, although modern microarray methods enable the use of very low volumes (<5 µL) and measurement parallelization (cell health assays) [63]. Supernatant measurements, either online or in-person, may provide measurements of nutrients and metabolites with little to no time lag. Tiny sensors that rely on optical detection using dyes are often utilized [64]. A thorough study of in situ dissolved oxygen measurements in OOC was conducted by Rivera et al. [65]. A crucial component of metabolism is dissolved oxygen, particularly in the action of the liver’s CYP450 enzyme [66]. Microprobes or pH-sensitive patches may be used to monitor pH and dissolved oxygen. Microscopy or optical fibers are required for optical detection and automated solutions [67]. Biosensors play a crucial role in organ-on-a-chip systems by enabling real-time monitoring of various biological parameters, which is essential for understanding cellular responses and evaluating the performance of these platforms [68]. Electrochemical enzyme-based biosensors test specific analytes, including lactate and glucose. These online, almost continuous observations are preferred since periodic measurements could exclude significant response variations. Analyte concentrations may be spatially resolved if sensors or patches are incorporated as arrays at close range or in direct contact with tissue structures [69,70].

Visual examination is the most popular way to test the functioning of cells and tissues in OOC devices since microscopy and high-content imaging are two of the most widely used analytical techniques in cell biology. Most OOC devices can be built to fit inside the imaging depth of common epifluorescence or confocal microscopes since they are made of optically transparent materials, including glass, PDMS, and thermoplastics. Thus, by using the organ-on-a-chip’s microfluidic capability to supply the required chemicals, it is possible to label the cells and tissues on-chip using fluorescent dyes or antibodies to evaluate cell viability or the expression of certain biomarkers [71]. In recent years, imaging sample preparation processes (cleaning, staining, etc.) and analysis have evolved and been modified to cope with complex, 3D multicellular structures. These techniques include confocal microscopy and light-sheet microscopy. High-content imaging can provide spatial and temporal information on the morphology of cells and tissues, which may be used to evaluate innovative treatments and aid in understanding disease pathogenesis more effectively. An optical window to the cells and a design specification that permits positioning and alignment of the OOC on a microscope stage are necessary for visualizing the OOC systems. The tissue model, preparation techniques (fixing, staining, and clearing), and microscopy must be perfectly matched to the methodology for image analysis and data processing to be successful [72].

Other ways to examine cell functionality are measuring cells’ force and electrical activity. Cantilevers detect the force generated by cells, whereas microelectrode arrays (MEAs) assess electrical activity in cells and tissues. Compared to the patch clamp, the MEA system is well suited for use with OOCs. Using the heart model as an example, conduction velocity, beat frequency, and field potential length estimations can be made using a 2D array of cells, making this approach a good approximation of the QT interval, the period from ventricular depolarization to ventricular repolarization, which can be measured by electrocardiography as well. Several systems, including the cardiovascular, neuromuscular, skeletal muscle contraction, and nervous systems, may benefit from MEAs [73]. In particular, microtunnels have been used to characterize the propagation of action potentials from presynaptic to postsynaptic chambers [74]. Several organ activities would not be possible without mechanical forces, such as the formation of contractile force by cells or tissues like the lung and heart. Cells are etched onto silicon cantilevers, and their deflection may be monitored optically or electronically in OOC devices, allowing for non-destructive, near-real-time measurements. A TEER-MEA (Transepithelial Electrical Resistance) cardiac chip is capable of multifunctional measurement due to the integration of MEAs and electrodes for TEER measurements during chip manufacture [75]. The integrity and permeability of any barrier tissue may be evaluated by transepithelial electrical resistance (TEER) (such as the gastrointestinal tract, the kidneys, and the BBB). TEER levels are linked to the function of several native barrier tissues in the body. The BBB is the most impermeable barrier, with values of 1500–8000 cm^2^, in contrast to the proximal tube in a kidney, which has a value of around 70 cm^2^. Values shown in models of organs and tissues should match biologically (in vitro). Tissue models may sometimes provide far higher results than physiological values, while results might be significantly lower in other circumstances. In these cases, the completeness of the model is crucial, particularly when the physiological system is composed of several cell types, but the model only employs a subset of them [76,77].

### 3.4. Standardization

Standardization ensures reproducibility within OOC systems, encompassing internal and external interfaces [42]. Internal interfaces, such as cell surface interactions and matrix composition, require standardized protocols to maintain consistency [42]. External interfaces involve connections to external instruments, compatibility with other OOC devices, and ensuring comparability of outcomes across different systems and laboratories [42]. Moreover, standardization extends to data outcomes, ensuring consistency in structure, format, and conditions for inputs to computational models, as well as facilitating translation to clinical and animal data [42]. 

## 4. Organ-on-a-Chip

OOC technology can simulate various organs, including the kidneys, lungs, heart, and liver, which are discussed below. All OOCs utilize microfluidics, biocompatible materials, and sensing components. These sensing components can include an automated imaging system, an embedded output sensing component, or various other microsensors [2]. 

### 4.1. Kidney-on-a-Chip

**Figure 1:**
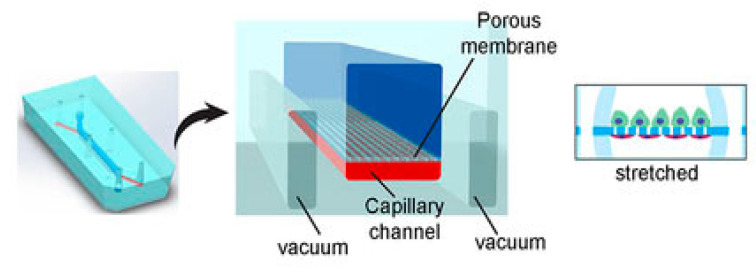
Kidney-on-a-chip design [80].

The kidneys remove wastes and extra water from the blood as urine and help keep the chemical balance of sodium, potassium, and calcium in the body. They also produce hormones that help control blood pressure and stimulate the bone marrow to make red blood cells. Jang et al.’s [78] group were the first to create a multilayered microfluidic system that simulated renal filtration using mouse kidney cells. The same device was later used to culture human kidney cells; this was among the first toxicity studies of primary kidney epithelial cells [79]. Musah et al. [80] created a kidney-on-a-chip that mimicked the structure and function of the capillary wall, as seen in Figure 1. Mechanical pressure can be applied to the cell layer via vacuum chambers on either side. Many other studies revolve around disease modeling, drug screening, and toxicology assessment. Kim et al. tested the nephrotoxicity of gentamycin in two different pharmacokinetic profiles using kidney-on-a-chip. They found that continuous infusion has less nephrotoxicity compared to bolus injection. They also suggested using microfluidic cell culture models for further pharmacokinetic and toxicity studies [81]. 

### 4.2. Lung-on-a-Chip

**Figure 2:**
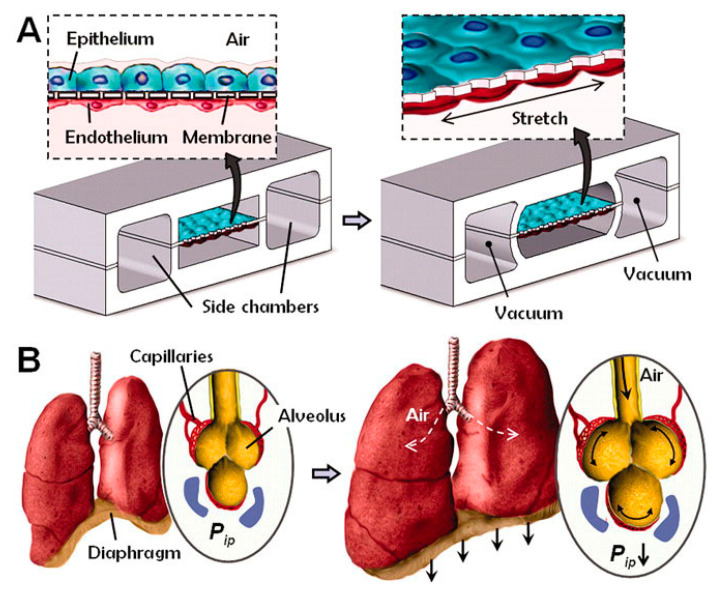
Huh et al.’s lung-on-a-chip. (**A**) The microfabricated lung device replicates breathing motions through vacuum-induced mechanical stretching of PDMS membranes, forming an alveolar-capillary barrier within the compartmentalized microchannels. (**B**) Diaphragm contraction lowers intrapleural pressure (P_ip_), stretching the alveolar-capillary interface during inhalation in living lungs [39].

Gas exchange in the human lungs is regulated by alveoli, tiny sacs that allow for the blood exchange of oxygen and carbon dioxide during breathing. This process has been challenging to reproduce in vitro. However, OOC technology has allowed researchers to create accurate lung microenvironments that sustain consistent gaseous exchange. Current studies focus on regulating mechanical airway pressure and the blood–brain barrier [82]. Huh et al. [39] created a lung microenvironment separated into regions, as shown in Figure 2. The upper region contained alveolar cells, and the lower region had pulmonary cells; this simulated the alveolar–capillary barrier. The system was operated in a vacuum to simulate the expansion and contraction of alveoli during respiration. Inflammatory stimuli were also introduced to the system as neutrophils through the fluidic channels. 

Blume et al. [83] designed a chip that simulated pulmonary interstitial flow utilizing a permeable filter and a single tissue culture chamber. Multiple chambers could be combined for improved integration. Pressure could be applied to the alveoli and attached capillaries, providing a shear flow profile and realistically simulating a lung microenvironment. Lung-on-a-chips are also useful as respiratory assistance devices. Another study on lung-assist devices allowed additional gas exchange in the placenta for unborn infants in the case of respiratory failure [84]. Dabaghi and others [85] utilized double-sided gas delivery on their microfluidic blood oxygenators to improve gas exchange. Oxygen uptake on the double-sided device increased by 343% compared to single-sided devices. 

### 4.3. Heart-on-a-Chip

**Figure 3:**
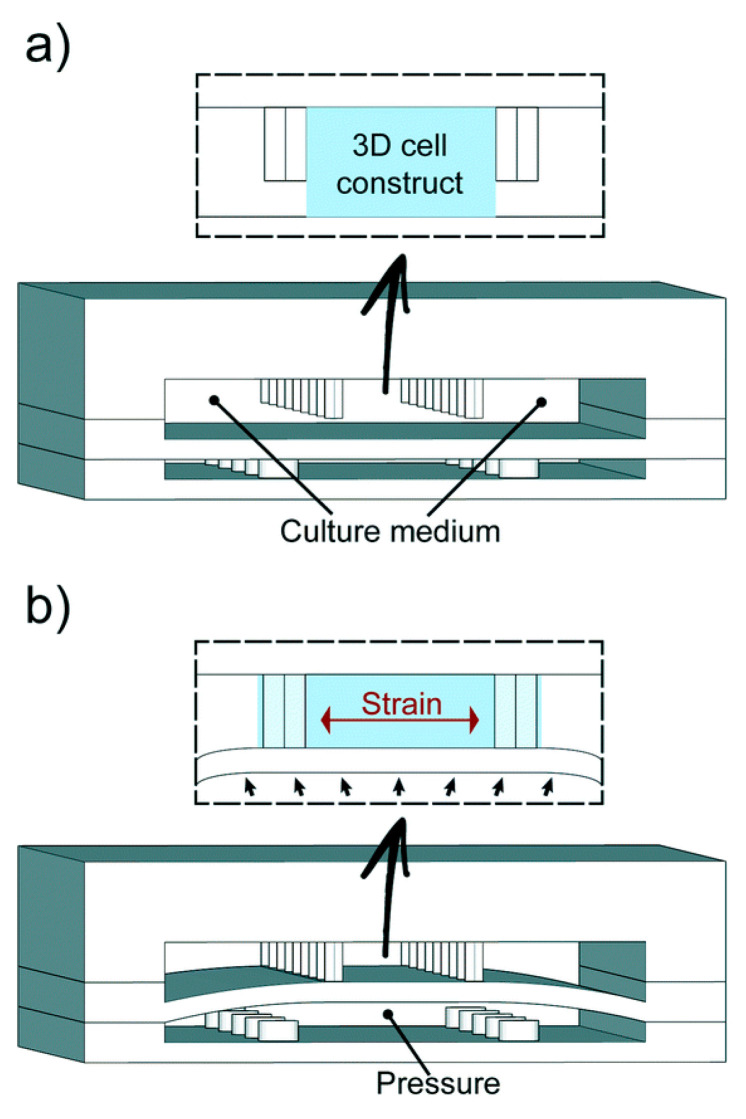
Marsane et al.’s heart-on-a-chip. (**a**) The microfabricated heart-like device has two PDMS chambers with cardiac cells in a fibrin gel matrix, replenished by side channels, allowing for configurable geometries. (**b**) Pressurizing the bottom compartment deforms the PDMS membrane, compressing the 3D cell construct against postsgenerating cyclic pressure to mimic physiological heart phases [87].

Microfluidics has enabled novel research on cardiac tissue. The myocardium is the heart’s muscle tissue; the anatomical unit of the myocardium is cardiomyocytes (CMs). Grosberg et al. [86] used a PDMS chip to implant rat CMs to form muscle membranes on a membrane. When the CMs contracted, the membrane curled, and the degree of the curl was measured to analyze cell contraction capabilities. A platform was designed by Marsano et al. [87] that mimicked the physiological and mechanical microenvironment of the heart using CMs, as seen on the right in Figure 3. The lower compartment is pressurized to deform the separating membrane and to compress the 3D structure, simulating the heart pumping. Other studies used CMs to directly assess the effects of various drugs related to the rate of the heart pumping [88]. There have been multiple advances in drug development because of drug trials in cardiotoxicity performed on heart-on-a-chips.

### 4.4. Artery-on-a-Chip

**Figure 4:**
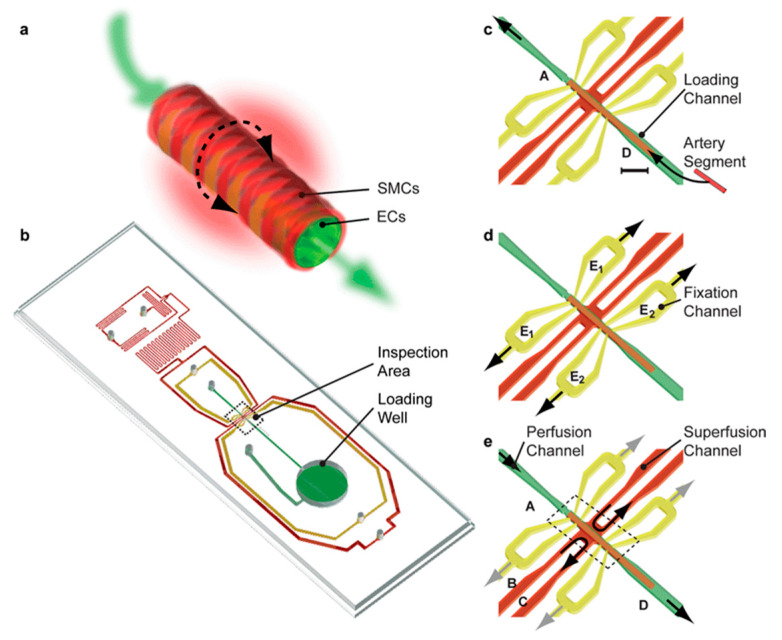
Gunther et al.’s artery-on-a-chip. (**a**) Illustration of a resistance artery segment. (**b**) Illustration of the chip housing a network of microchannels, a loading well for arteries, and an inspection area for arteries. (**c**–**e**) Illustrations of reversible procedures for artery segment loading, fixation, and inspection [56].

High blood pressure causes heart disease, stroke, and other conditions. Tiny artery pathologies cause and sustain cardiovascular disease. Analyzing tissue slices, single and co-cultured cell populations, and bioengineered tissues with microfluidics can reveal underlying mechanisms. Better treatment procedures require scalable ways to study intact cardiovascular tissues in health and sickness [89]. Most existing technologies perfuse small arteries with glass micropipettes, requiring skilled expertise and often not scalable. Günther et al. [56] created a scalable organ-based microfluidic platform to load, position, fix, perfuse, and superfuse a frail resistance artery segment. Resistance arteries regulate organ blood flow and redistribution. Endothelial cells (ECs) and smooth muscle cells (SMCs) make up the terminal of the arterial vascular tree. ECs can produce vasoconstricting and vasodilating chemicals to control vascular tone. The platform featured a loading space, a microchannel network, and an inspection area. A thermoelectric heater and thermoresistor maintained the artery inspection region at 37 °C. Yellow microchannels were used for arterial fixation and perfusion, and red microchannels were used for superfusion, as shown in Figure 4. A segment of the resistance artery was immersed in the loading well to begin the artery loading process. Fixation and superfusion inlet and outlet lines remained closed. The loading well remained open, and microchannel A was connected to a syringe pump dispensing a constant flow of buffer, transporting the artery segment to its dedicated position in the inspection area. Yellow microchannels apply sub-atmospheric pressure to the artery segment. The loading well mimics arterial blood supply to a capillary. The last pair of red microchannels maintain organ model physiology and metabolism with superfusion flow rates and a stable sustaining medium. This gadget can study artery structure and function in a well-defined spatiotemporal environment. According to the platform, the vasoconstrictor response did not propagate to the contralateral side [90]; adding phenylephrine to one or two superfusion channels spatially confined the no-stimulated side. This scalable experimental platform could identify, validate, create, and test cardiovascular drugs. Another study created an artery-on-a-chip to explore two cardiovascular diseases: carotid artery disease (CAD) and abdominal aortic aneurysms (AAAs). Both diseases share common characteristics of vessel wall remodeling and changes in hemodynamic shear stress. The chip consists of a membrane layer and two glass slides that can be resealed, forming the upper and lower chambers of the artery-on-a-chip device. Flow sensors were strategically placed between the reservoirs and the chip to ensure consistent flow rates. The cells used in this study were primary human aortic endothelial cells (ECs) and human aortic smooth muscle cells (SMCs). 

### 4.5. Liver-on-a-Chip

The liver is the leading site of drug/toxin metabolism. It comprises anatomic units called hepatic lobules; these hepatocytes work together to filter the blood from the digestive tract by detoxifying chemicals and metabolizing drugs [91]. The first liver-based microfluidic system was designed by Kane et al. [92], consisting of rat liver cells and mouse fibroblasts cultured to mimic an airway interface. Rat hepatocytes were cultured in the chip and were shown to synthesize and metabolize albumin stably. A chip established by Lee et al. [92] imitated the interstitial space between liver endothelial cells and hepatocytes, facilitating new opportunities for substance exchange studies. Using radical electric field gradients and electrophoresis, Ho et al. [93] restored the hexagonal shape of the hepatic lobule that allows cell arrangement onto a circular PDMS chip. Chong et al. [94] were able to scrutinize the metabolism process via assays that keep track of skin reactions to drugs. This study can impact drug screening research as it can identify compounds that produce systemic skin reactions. Several studies involving disease/injury research, such as Kang et al. [95], analyzed the replication of hepatitis B, and Zhou et al. [96] modeled alcohol injury using a liver OOC. It is crucial to obtain further knowledge regarding hepatitis B since more than 240 million people worldwide are infected with HBV, which may cause liver cancer. Noh et al. found that HBV infection was induced by transfecting HBV-genome cDNA onto a microfluidic platform and infecting the cells with an HBV genome expressed from a recombinant adenovirus. For HepG2 cells, transfection had a high infection efficiency; for primary rat hepatocytes (PRHs), adenovirus infection had a higher infection rate [97]. Table 2 demonstrates materials, sensors, and types of cells commonly used in liver-on-a-chip systems.

### 4.6. Intestine-on-a-Chip

**Figure 5:**
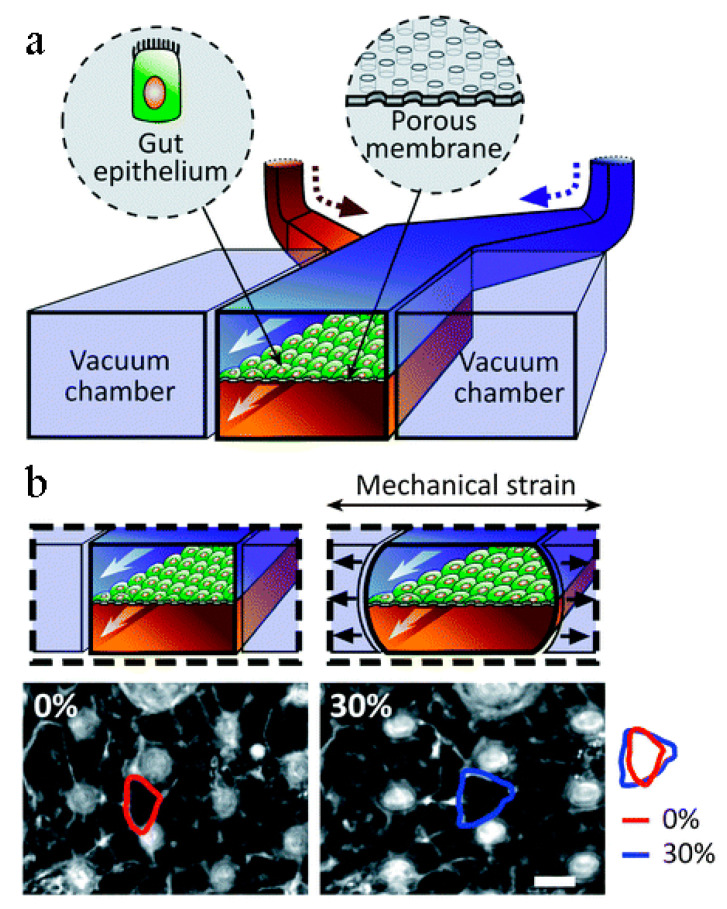
Kim et al.’s intestine-on-a-chip. (**a**) Schematic of a gut-on-a-chip device: flexible ECM-coated membrane with gut epithelial cells spans central microchannel, flanked by full-height vacuum chambers. (**b**) Images show intestinal monolayers in gut-on-a-chip: with (right) or without mechanical strain (left) (30%; arrow indicated direction); suction applied to vacuum chambers causes cell distortion toward tension direction [44].

Similar to the lungs and their alveoli, the human small intestine is lined with small appendages called villi that are key to the adsorption of nutrients. Current studies involving intestine-on-a-chip include the adsorption of oral drugs into the bloodstream and a general analysis of microbial flora found in the gut. Kim et al. [44] created an intestine-on-a-chip by constructing a chamber bilayer separated by a porous membrane lined with gut epithelium cells, as seen in Figure 5. Drugs or inflammatory cells were introduced to the bottom chamber to analyze adsorption and cell reaction. Two vacuum chambers on either side of the cell bilayer simulated the cyclic strain that a human small intestine involuntarily creates. This study demonstrated the potential of intestine-on-a-chip for personalized medicinal studies. Kasendral et al. [102] developed a model of the human duodenum, the first part of the human small intestine, on a microchip to enhance the knowledge of the microbiome environment within the gut and intestinal morphology. To study the human gut microbiome, Firouzinezhad et al. co-cultured aerobic and anaerobic microbiota in the intestine-on-a-chip, which enabled them to control and assess the oxygen gradient. This platform may be a future discovery tool for microbiome-related therapeutic studies [103]. 

### 4.7. Brain-on-a-Chip

The brain is a complex organ that includes a variety of functional units and cell types, and each is responsible for a different task. The aim is to model such an organ in a microchip to reduce the cost and time used to test certain drugs that correlate to central nervous system (CNS) diseases. Modeling a brain on a chip that incorporates all aspects of brain functionalities is nearly impossible due to the different cell culturing needed in the same system. Therefore, the brain-on-a-chip needs to be designed with a unique platform for modeling only one specific compartment of the brain [104]. According to Dr. Maoz, there is no “one brain-on-a-chip fits all” model, and each application requires a different platform, which may require different considerations in terms of practicality. Researchers interested in developing and using such a platform should first consider the problem they are trying to solve, define the most critical parameters that might affect the result, and identify which platforms will allow them to incorporate the parameters of interest [104]. Some of the materials, sensors, and cell types explored by researchers in designing brain-on-a-chip models are listed in Table 3.

The blood–brain barrier is a massive challenge in drug development for neurological diseases, and animal models do not provide enough information about the mechanisms of BBB function and their interaction with the therapeutic. Researchers mimicked the BBB on a chip to study the interaction between the BBB and a nanoparticle at the cellular and molecular levels [105]. 

Research conducted by Tong et al. used a microfluidic approach to study the regeneration of the axons after axotomy, which is essential for patients who suffer from neural injuries. Using this approach, they experimented with mimicking the in vivo microenvironment in the chip and exposed the axons to the inhibitory molecule after axotomy to observe the axon’s reaction [106]. Another process in the CNS is myelination, which forms a myelin sheath around the axons to enable neurons to transmit signals faster and more efficiently. A study applied a microfluidic device to examine different stages of myelination using embryonic stem cells derived from neurons [107]. 

Despite advances in disease modeling and the development of effective treatments, the exact pathological mechanisms of the initiation and progression of Alzheimer’s disease remain unclear. Studies have revealed that Alzheimer’s disease is caused by an abnormal accumulation of amyloid-β (Aβ) and the hyperphosphorylated microtubule-associated protein Tau. Microfluidic devices have been used recently to gain new insights into the trafficking and accumulation of pathological Aβ and Tau in human cells [108].

**Table 3:** Various brain-on-a-chip materials, sensors, and cell types.

Material	Sensor	Cell Type	References
PDMS	Electrical	hCMEC/D3 human brain endothelial cell line	[109]
PMMA	Electrical	bEnd.3 murine brain endothelial cell line	[109]
Polyethylene terephthalate and PDMS	Electrical	Human iPSC-derived endothelial cells, human astrocytes	[110]

### 4.8. Nerve-on-a-Chip

Experimental models of neurodegenerative illnesses have not been successful in predicting outcomes since a large number of clinical trials are unsuccessful, and less than 7% of neurological medications are commercially available [111]. Because of the complexity of the nervous system and the interaction between neurons and glia in the brain, micro-physiological systems used to assess possible human responses to novel treatment options lag behind those used to evaluate reactions in other organs. There are inadequate human-relevant in vitro models, particularly for peripheral nerves [112]. Animal research remains the fundamental basis since it is the only research that validates the histopathology and electrophysiology of a complex biological system, which are the most often used clinically significant measures for evaluating peripheral neurotoxicity [113]. Utilizing in vitro neural cells, nerve-on-a-chip models have recently been created to simplify the complexity observed in vivo. Microfabrication is used to create in vitro extracellular recording interfaces to track action potentials, ensuring reproducibility and enabling statistically significant sample volumes. Planar microelectrode arrays or microchannel electrodes integrate axonal transmission with high signal-to-noise ratio (SNR) recordings to form nerve-on-a-chip models. High-density complementary metal-oxide-semiconductor microelectrode arrays with a built-in microfluidic channel could assist in the detection of complicated impulses along individual axons at the microscopic level [114]. A recent study by Sharma et al. [115] designed a three-dimensional functional human peripheral nerve in vitro using the nerve-on-a-chip platform. They effectively created human neurons and primary human Schwann cell spheroids, which showed long-term cultures and robust neurite outgrowth in the dual-hydrogel system. This was the first biomaterial micro-physiological system of the myelinated human peripheral nerve that could be utilized to evaluate electrophysiology, which was previously only achievable with in vivo experiments. 

### 4.9. Pancreas-on-a-Chip

Treatment with insulin has been recognized as a gold-standard therapy for Type 1 diabetic patients. However, this method cannot precisely mimic the effects of normal beta cells and possible hypoglycemic attacks [116]. Thus, pancreas beta cell transplantation and islet replacement therapy were proposed as effective treatment techniques for insulin-dependent diabetes. Existing approaches for beta cell transplantation include separating and purifying the islets from the rest of the pancreatic tissue, frequently resulting in ischemia damage and an inflammatory signature response [117] and evaluating the quantity and quality of islet cells before transplantation is vital. Pancreas-on-a-chip (POC) is a technology that enables researchers to evaluate islet potency and quality using a standardized platform. This platform has been demonstrated to be used as a tool for islet clustering to investigate the islet function in a standardized situation [118]. Several POC systems were used in a microfluidic environment to evaluate the pancreas islet cell function in specific pancreas dysfunction. In a study by Shik et al. in 2019, POC was utilized to evaluate islet cell function in cystic fibrosis [119]. 

### 4.10. Placenta-on-a-Chip

The placenta is essential for a pregnancy to be successful. The primary role is the interchange of endogenous and external molecules, which permits appropriate oxygen and nutrition delivery, excretion of fetal metabolic waste, and defense against potentially dangerous agents. A multilayered structure commonly referred to as the “placental barrier, “made up of trophoblasts, connective tissue, basal lamina, and the fetal endothelium, allows substances to be transferred between the intervillous space and fetal capillaries [119]. Several factors, including umbilical blood flow, metabolism, thickness, etc., can influence placental transfer. OOC technology can help researchers to evaluate placental transport using microfluidics and microfabrication. Placenta-on-a-chip enables researchers to build a physiological placental barrier in vitro using human trophoblasts, umbilical vein endothelial cells, and extracellular matrix membranes. This technology might solve the primary drawbacks of existing placenta model systems, such as three-dimensional organoids from human blastocysts, and provide research platforms for reproductive biology and medicine [120].

### 4.11. Skin-on-a-Chip

In vitro and ex vivo dermatological, pharmacological, and cosmetic investigations use artificial membranes from rebuilt skins or excised skin samples. Skin permeability, irritation, corrosion, toxicity, and different disease models can be evaluated through reconstructed skin tissue. Also, different formulations and pharmacologic features of different drugs could be tested in these environments. In the literature, many skin-on-a-chip platforms have been proposed, including epidermal and full-thickness skin models as well as diffusion surface materials (membranes—STRAT-M^®^, chitosan, parallel artificial membrane permeability assay (PAMPA), skins of both animal and human origin) [121]. Dr. Risueno and his colleagues classified devices based on how the skin is generated on the chip. In designing microfluidic chips for modeling the skin, two major approaches have been developed: the first is the direct introduction of a skin fragment from a biopsy or a human skin equivalent (HSE) directly into the chip (transferred skin-on-a-chip), while the second is the generation of tissue on the chip in situ (in situ skin-on-a-chip) [122]. The transferred skin-on-a-chip model uses two primary sources of tissue fragments: a skin biopsy obtained from a donor or an in vitro-generated HSE. Both laboratory-made and commercially available HSEs have been used for skin microfluidic chips. Among HSEs, the ones with a dermal compartment are most commonly used for transferred skin-on-a-chip. As a result, more realistic models can be created of the various layers of skin present in these well-formed tissue fragments. The in-situ skin-on-a-chip model focuses on skin generation on the chip, and it can also be divided into two groups. Jones et al. (2022) developed a unique skin-on-a-chip model incorporating human-derived primary and immortalized cells in a full-thickness skin analog. The model was contained in a microfluidic device with a previously built microvasculature. This genuine microvasculature could help evaluate the efficacy and safety of medications administered systemically to patients [123]. It was shown by Kim et al. (2022) that human skin analogs might be utilized to simulate the main pathogenic aspects of atopic dermatitis, overcoming the shortcomings of prior studies that have relied primarily on mice models and have been unable to extrapolate their effects to people [124].

### 4.12. Stomach-on-a-Chip

Existing experimental laboratory approaches and animal models have substantial limitations for mimicking human gastrointestinal disorders since they might not fully depict multicellular human primary tissues. Consequently, new medicines must be validated using model systems that mirror human in vivo development, physiology, and disease processes. The production of three-dimensional (3D) human gastric organoids (hGOs) by guided development of human pluripotent stem cells was an important step toward this objective. Normal stomach activities and illnesses occur in the luminal epithelium; however, reaching the epithelium on the interior of organoids is difficult [125]. Lee et al. [126] described a novel microfluidic imaging device with peristaltic luminal flow that houses hGOs in vitro. This model enables stable, long-term 3D development of hGOs with luminal delivery capability through a peristaltic pump. Organoids were cannulated, and the fluorescent dextran-containing medium was supplied through the lumen with a peristaltic pump. This method also enabled the researchers to rhythmically add stretch and contraction to the organoid, simulating gastrointestinal motion. This technology can transport nutrients or pharmacological substances into the gastric lumen in vitro to study human physiology, disease modeling, and drug screening. Li et al. presented an in vitro microfluidic tool to study mucin secretion against acidic digestive juice and the protection of epithelial cells in the stomach. Their result demonstrated that the mucin secretion layer inhibits acid diffusion. This system may be beneficial in studying mucus–drug interactions [127].

### 4.13. Muscle-on-a-Chip

Skeletal muscle is mainly responsible for generating contractile forces and ensuring the functional performance of critical functions, including mastication and eye movements [128]. Tissue-engineered skeletal muscle offers a potentially valuable option for physiologically relevant testing and study of the musculoskeletal system. For example, these tissues could be used in vitro to study how the human musculoskeletal system responds to injury, disease, and treatment [129]. The construction of a three-dimensional (3D) skeletal muscle tissue that mimics the morphological and structural intricacies of muscle inside a microfluidic device was described in a study by Agrawal et al. [130]. Using a 3D photo-patterning technique, scientists spatially confined a cell-filled gelatin network around two bio-inert hydrogel pillars, which support the linear elastic alignment of the cells and act as attachment points for the encapsulated cells and muscle tissues as they develop and expand. The dose-dependent impact of cardiotoxin on the designed muscle tissue architecture was investigated, as was the subsequent effect of the cardiotoxin on the passive tension [130]. Studies that need to analyze the structure and function of skeletal muscle, such as preclinical drug discovery and development, may find this tool intriguing because of its simplicity while maintaining its high level of effectiveness.

### 4.14. Testis-on-a-Chip

Testis-on-a-chip investigations bring up new avenues for the analysis and implementation of in vitro testicular function. They may also facilitate incorporating microfluidic technology into future treatment techniques to maintain prepubescent male fertility and maturation arrest in adults. Finally, they may offer an environment close to the human body for evaluating drugs and toxins [131]. For the advancement of in vitro spermatogenesis, Abu Madighem et al. [132] brought up new possibilities for a microfluidic 3D culture system, a testis-on-a-chip platform. From the seminiferous tubules of sexually immature mice, testicular cells were enzymatically extracted, seeded in a methylcellulose gel, and grown on a microfluidic chip. The cells continued to divide and differentiate as they grew into spheroids. Over 95% of the cells were still viable after seven weeks of cultivation. A structure that includes premeiotic, meiotic, and post-meiotic germ cells, representing each step of spermatogenesis up to and including meiosis II, was shown on the platform. The Sertoli and peritubular cells that serve as supports were also present in the spheroid formation. The experiment also investigated how sensitive the system was to the culture medium’s addition of retinoic acid and testosterone. A traditional 3D methylcellulose cell growth system in a well plate was contrasted with the testis-on-a-chip as a standard. Fluorescence-activated cell sorting analysis revealed more haploid cells on the chip than in the plates. Immunofluorescence labeling after seven weeks of culture revealed that the chip had more differentiated cells than the well plate. 

### 4.15. Bone-on-a-Chip

All three types of bone cells (osteoblasts, osteocytes, and osteoclasts) differentiation, activation, and apoptosis frequently depend on intracellular communication between them, which occurs via ligand–receptor interactions, molecules and ions moving via the extracellular matrix (ECM) and gap junctions [133,134]. Riquelme et al. created a microfluidic technology for researching bone cell–cell contact at physiologically significant distances. This platform examines how osteoclasts and osteocytes interact to cause pathogenic bone illnesses such as osteoporosis and metastasis. Two or three channels are separated by high-resistance posts making up the microfluidic platform. Additionally, bone-on-a-chip is used for tissue engineering, vascularization, and bone regeneration. The bone-on-a-chip platform’s relative newness compared to other organ-on-a-chip systems makes investigations of bone cancer metastasis rare. The role of bone-on-a-chip is still being investigated.

### 4.16. Bladder-on-a-Chip

The bladder-on-a-chip model contains critical physiological elements for detecting early uropathogenic Escherichia coli (UPEC) infection on a pressure-driven flow control platform. This platform is suitable for live-cell imaging and antibiotic delivery. A layer of bladder cells on the chip created by Sharma et al. sits at the bottom of a channel filled with diluted human urine. Infected cells are scanned to see how UPEC migrates, interacts with bladder cells, and aggregates [135]. In addition to infection detection, a bladder-on-a-chip system has been used for noninvasive diagnostic purposes. Wang et al. developed a protein microarray chip to capture bladder cancer cells and biomarkers from urine samples [136]. Similarly, Lin et al. measured biomarker concentration quantitatively using a bead-based enzyme-linked immunosorbent assay [137]. Moreover, other research groups developed a microfluidic system that can recognize bladder cancer cells at high processing speeds, which serves to study bladder cancer pathophysiology [138]. 

### 4.17. Eye-on-a-Chip

Choroidal neovascularization (CNV) results in the localized breakdown of the outer blood-retinal barrier, which leads to various retinal diseases [139]. Eye-on-a-chip (EOC) technological advancements have facilitated the successful recapitulation of the CNV pathogenesis [139]. Notably, EOC platforms have been useful in modeling the dose-dependent effects of bevacizumab, a clinical CNV treatment, in inducing the penetration of angiogenic sprouts from preexisting choroidal vessels and the subsequent breakdown of the retinal pigment epithelial (RPE) monolayer [140]. Moreover, another retina-related EOC model has shed light on the impact of alterations in glucose concentration and oxygen levels on the secretion of vascular endothelial growth factor (VEGF) by retinal pigment epithelial cells (ARPE-19) [140]. Additionally, blinking EOC models interfaced with human-scale diagnostic tools have been developed to simulate evaporative dry-eye disease, facilitating drug testing and discovery efforts [140].

### 4.18. Spleen-on-a-Chip

The spleen is a secondary lymphoid organ that filters out senescent, damaged, or infected red blood cells [141]. This capacity is primarily because of the microcirculation through filtration beds within the splenic red pulp in an open-slow microcirculation compartment. To replicate the spleen’s filtering function on a chip, researchers have developed a novel micro-engineered device that mimics the hydrodynamic forces and physical properties of the splenon [141]. Using human red blood cells and malaria-infected cells, they assessed the mechanical and physiological responses of the splenon within the chip [141]. 

The spleen may not always sufficiently eliminate pathogens, especially in individuals who are ill or have suffered traumatic injuries, potentially leading to life-threatening sepsis [142]. To prevent such outcomes, a microfluidic artificial spleen device has been developed to purify the blood of sepsis patients. In this device, magnetic nanoparticles have been utilized and modified with mannose-binding lectin (MBL). This human blood opsonin binds to a wide range of pathogens, including bacteria, viruses, parasites, fungi, and toxins [142]. 

### 4.19. Bone-Marrow-on-a-Chip

Bone marrow contains hematopoietic stem cells that can become red blood cells, white blood cells, or platelets. It also contains stem cells that can become cartilage, fat, or bone cells. Bone-marrow-on-a-chip sustains these types of stem cells and preserves the spatial structure within an in vitro-developed 3D bone marrow niche. These characteristics of the bone-marrow-on-a-chip are likely essential to its capacity to sustain the intricate functionalities of the entire organ, which are incomparably difficult for traditional stroma-supported cultures to imitate. Notably, microfluidics also makes it possible to analyze responses under flow, which is crucial for controlling bone marrow physiology [143,144]. The bone-marrow-on-a-chip offers an intriguing alternative to animal models because it allows for the in vitro manipulation of specific hematopoietic cell populations (either genetically or through the use of drugs) as well as the insertion of different cell types (such as tumor cells) before examining how the intact marrow responds to relevant clinical challenges, such as radiation or medications. Finally, bone-marrow-on-a-chip is a potent technique for speeding up research and development in various biomedical domains, including hematology, oncology, drug discovery, and tissue engineering [145].

### 4.20. Uterus-on-a-Chip

**Figure 6:**
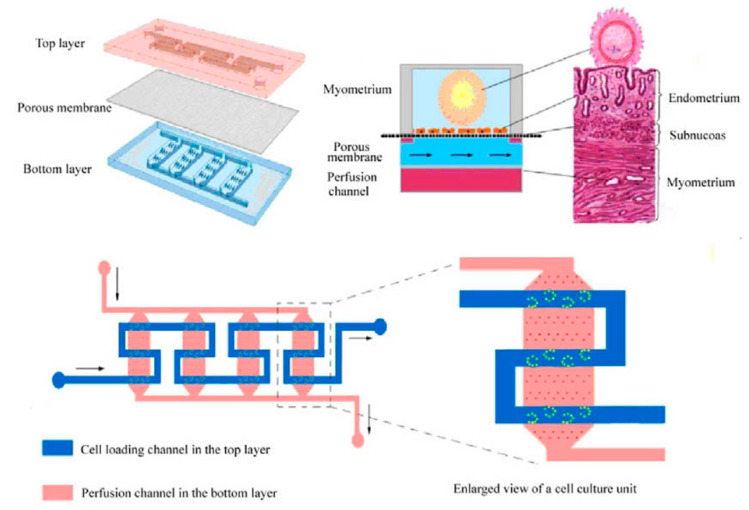
Uterus-on-a-chip [146].

The female reproductive system is developed at puberty and consists of the vagina, cervix, uterus, fallopian tubes, and ovaries. Each releases hormones that communicate with other parts and regulate the female body, including the organized menstrual cycles. During pregnancy, the uterus is where the eggs are fertilized after passing from the ovaries through the fallopian tubes. Being able to design a part or all of the female reproductive system on a chip can lead to a better understanding of physiology. In turn, it could encourage new methods of approaching medical cases related to the female reproductive system. In vitro fertilization–embryo transplantation (IVF-ET) is a process of fertilizing the egg out of the female body and is one of the widespread treatments for infertility. However, IVF-ET has some limitations, such as a low feralization rate. To tackle these limitations, a microfluidic uterus is developed to perform processes in an embryo-like environment, including ovulation, insemination, and embryo development. As shown in Figure 6, the microchip consists of three layers: Two PDMS layers are separated by a porous layer used to support the endometrial cell culture. The top layer had a zigzag-shaped channel and several interlaced microsievers on the channel bed to capture oocytes. In contrast, the bottom layer is designed with four parallel channels with a micropillar array on each bed to support the porous membrane [146]. 

### 4.21. Other Applications of OOCs

#### 4.21.1. Cervix-on-a-Chip

A solid alternative to the in vitro model for studying the physiology of the cervix in real-time using high-resolution imaging for analysis of biological reactions in the cervix, as well as medication discovery, is modeling via a uterine cervix-on-a-chip. The well-established uterine cervix-on-a-chip is transparent, efficient, and simple to use. It is anticipated to have significant implications for the customized management of human papillomavirus infection lesions and cervical cancer, as well as a possible role in tissue engineering and other clinical treatments [147]. A different study showed that cervix-on-a-chip is effective for obstetrics and gynecological research. It can be used to study the dynamics of cervical epithelial cells in the benign or malignant cervix and the remodeling of the cervical cavity during pregnancy and parturition [148]. 

#### 4.21.2. Hair-on-a-Chip

Ataç et al. cultured ex vivo prepuce as a skin organ culture to evaluate the native state of the tissue. They compared their results in two different media culture conditions. In the first condition, they cultured the cells in Transwells (static culture), while in the second, they used a dynamically perfused microchannel system (multi-organ-on-a-chip). The results from their study showed a distinct difference between the parallel static and multi-organ-on-a-chip (MOC) cultures and that dynamic perfusion prevents tissue disintegration. MOC hair follicle cultures revealed many hair fiber extensions from the epidermis, while there was triple the culture period compared to the Philpott assay (using single, truncated hair follicles to study hair follicle biology in vitro) for ex vivo hair elongation and drug detection. These findings demonstrate that hair-on-a-chip may evaluate hair follicle biology [149].

#### 4.21.3. Lymph-Nodes-on-a-Chip

Lymph-nodes-on-a-chip are appealing platforms for examining immune-system-related illnesses like cancer and inflammatory diseases. Biomimetic lymph-nodes-on-a-chip systems can load healthy or diseased cells or tissues, and their behavior can be easily observed. Treatment regimens that use immunosuppressive medications to target the immune system for treating certain illness conditions greatly benefit from lymph-nodes-on-a-chip technology. Immunotherapy has long been used to treat allergies, lessen the chance of rejection of transplanted organs, and minimize autoimmune disorders [150]. Additionally, it is now a typical kind of treatment for many tumors. Numerous solid and hematologic cancers have responded well to cancer immunotherapy, and it is well-known that lymph node draining is helpful [151]. Additionally, it has been demonstrated that immunotherapies have distinctive toxicity profiles linked to particular modes of action [152]. Lymph-nodes-on-a-chip platforms that are physiologically appropriate can be used to explore these phenomena extensively. Lymph-nodes-on-a-chip are also helpful in studying immune-related disorders like cancer and inflammation [153]. Biomimetic lymph-nodes-on-a-chip devices can easily monitor normal or sick cells or tissues [153].

#### 4.21.4. Vagina-on-a-Chip

**Figure 7:**
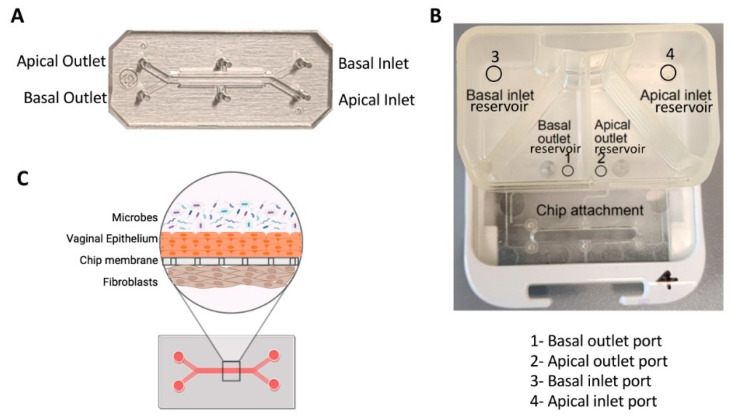
Vagina-on-a-chip. (**A**) Image of a two-channel PDMS chip. (**B**) Image of a pod demonstrating the reservoirs for basal and apical channels. (**C**) Illustration depicting a cross-sectional view of a Vagina Chip infected with microbes [155].

Mahajan et al. developed a vagina-on-a-chip model to improve our understanding of the interaction between the host and microbiome in the vagina. They co-cultured primary human vaginal epithelial cells and uterine fibroblasts in the microfluidic device to mimic the vaginal epithelial–stromal interface. Co-culturing with *Lactobacillus crispatus* containing consortia maintained epithelial cell viability and caused a physiologically low pH, D-lactic and L-lactic acid accumulation, and downregulation of inflammatory cytokines. However, co-culturing with Gardnerella vaginalis-containing consortia led to injury in epithelial cells, increased pH, and upregulation of pro-inflammatory cytokines [154]. A recent study utilized human vagina-on-a-chip to co-culture human vaginal microbial consortia with primary human vaginal epithelium, to investigate the physiological responses of the human vagina to healthy and dysbiotic microbiomes [155]. The chip had parallel hollow microchannels hosting living cells under dynamic fluid flow [155]. These chips facilitate the recreation of organ-level tissue–tissue interfaces by culturing diverse cell types, such as epithelium and stromal fibroblasts or epithelium and vascular endothelium, on opposite sides of a porous membrane within channels. Figure 7 demonstrates the design of the chip [155].

### 4.22. Multi-Organs-on-a-Chip

**Figure 8:**
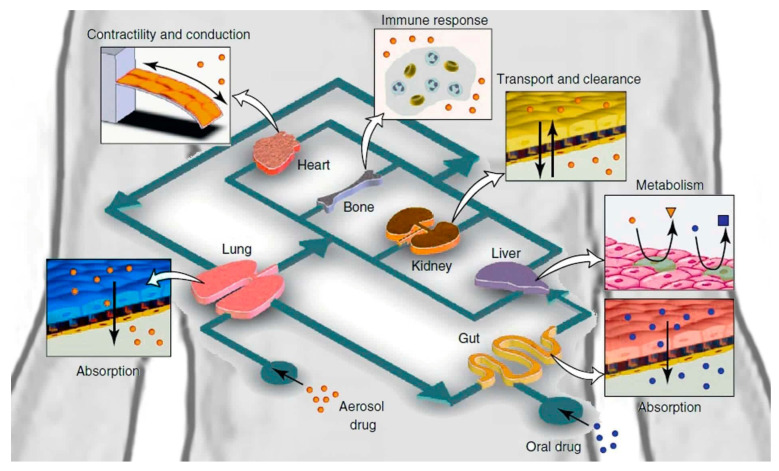
Diagram of human-on-a-chip. This image was taken from the ELVESYS Group website (elveflow.com).

The human body is complicated, and single-organ chips cannot fully and accurately reflect a human organ without recognizing that organ’s interactions with other organs and systems within the body. “Multi-organs-on-a-chip”, also known as the “human-on-a-chip”, is an ongoing research project aiming to simultaneously construct multiple organs on a single chip, as seen in Figure 8 [156]. Multi-organ integration will theoretically be achieved with different organs and tissues connected by bionic blood channels [157]. This will permit the analysis of whole-body system interactions. Ongoing studies include the design of two-, three-, four-, and even ten-organ chips. Midwoud et al. [158] successfully combined the liver and intestines on a microfluidic chip, to examine the regulation of bile acid synthesis. Lee and colleagues [2] developed pumpless, user-friendly multi-organs-on-a-chip, which were straightforward to assemble and operate. Satoh and colleagues described a multi-throughput, multi-organ-on-a-chip system created on a microplate-sized pneumatic-pressure-driven medium circulation platform [2]. Inevitably, the more organs added to a chip, the higher the complexity and the greater the challenge of controlling the system’s parameters. Overcoming these challenges would revolutionize the drug discovery research field. 

In terms of drug safety, there are various challenges to overcome. Two-dimensional cultures and animal studies are not truly representative of the human body. The liver metabolizes an administered drug, and the resulting compound may cause unpredictable toxicity to organs not intended to be affected, such as the heart. Several liver and heart models have been widely used to assess the toxicity of new or recalled drugs. A multi-organs-on-a-chip platform provides an innovative approach to studying drug-related effects, resulting in predictions of liver metabolism on off-target organs, ultimately improving drug safety testing during preclinical stages of the development process [159].

## 5. Disease Models on the Organ-on-a-Chip

In recent years, organ-on-a-chip technology has emerged as a promising platform for modeling and studying diseases in a physiologically relevant and accurate manner. Traditional methods to study disease states have relied on animal models and 2D cell cultures, which often fail to reflect the complexity of the human body. However, OOC technology has enabled researchers to recreate the microenvironment of multiple organs in order to develop more realistic disease models. The table presented below (Table 4) provides a summary of disease models on the organ-on-a-chip platform. 

## 6. Commercialization

During the translation and commercialization of organ-on-a-chip, the industrial perspective is prioritized, including technical standardization and dependability, simplicity of use, cost-effectiveness, and compliance with legal requirements [165,166,167]. Therefore, it is necessary to conduct additional analytical validations of these chips to evaluate their diagnostic and therapeutic efficacy and repeatability and to satisfy the practical requirements of pharmacological and medical use and FDA regulations. During each stage of organ-on-a-chip development, early and close collaboration between academic institutions, industrial R&D divisions, and healthcare organizations will create a positive feedback loop, which serves to confirm the efficacy of these platforms and maximize their utility in the healthcare industry. For instance, Emulate Inc., (Boston, MA, USA) has collaborated with AstraZeneca, Johnson & Johnson, Merck, Takeda, and the FDA to analyze the efficacy and safety of drug candidates for human use in an industrial setting, serving to validate the effectiveness of their various products.

Despite its vast potential, a significant challenge to the commercialization of organ-on-a-chip technology is insufficient venture capital funding. Only a few OOC startups, including Emulate Inc. (USD 142.3 M), InSphero (USD 35.2 M), and Mimetas (USD 34.2 M), have managed to secure substantial investments, according to public sources like Crunchbase. To overcome this challenge, national healthcare and research systems can play a vital role by fostering collaborations with academic institutions and companies, offering support for intellectual property protection, and providing funding avenues [168].

## 7. Conclusions and Recommendations

This paper has covered the on-a-chip research into the kidneys, lungs, heart, liver, intestines, brain, and uterus, but microfluidic technology has also been used to reconstruct other organs, including blood vessels, skin [169], skeletal muscle [170], and the central nervous system [171]. As such, OOC platforms are becoming increasingly valuable for studying the human physiology. These innovative endeavors have successfully recreated in vivo-like conditions in vitro, enabling the investigation of crucial factors such as cell blood flow, biochemical signals, and various cellular interactions that impact cellular behaviors [172].

While OOC technology continues to advance rapidly, challenges remain on the path toward achieving the “human-on-a-chip” concept. The need for a universal cell culture medium and the practicality of sample collection on such a platform present obstacle, worsened by the increasing complexity of each additional organ integrated into the chip. Addressing these challenges will require innovative solutions and ongoing research efforts.

One area of focus for improvement lies in the materials used for chip fabrication, with a need for alternatives to PDMS that more accurately mimic in vivo tissue properties. Additionally, cost-effectiveness and ease of use are crucial for widespread adoption, highlighting the importance of developing lower-cost, disposable components and reusable expensive elements.

In conclusion, while OOC technology has already yielded significant biological research advancements, its continued evolution holds the promise of further improving the accuracy and cost-effectiveness of microenvironments. Solving the challenges ahead will pave the way for transformative developments in biological systems research.

## Figures and Tables

**Table 1:** Choice of materials used in microfluidic applications.

Material	Property/Advantages	Disadvantages	Application	References
Collagen	Biocompatibility, control of the structure	Lacks mechanical strength and structural stability when hydrated	Biosensing, film assembly	[13,14,15]
Agarose Hydrogel	Biodegradability, mechanical stability	Poor biodegradable Poor cell adhesion Inconsistent properties	Cell culture, sensors	[16,17,18,19]
Teflon	Ease of fabrication	Hydrophobicity	Ultra-clean tools, valves, and pumps	[20]
Photocurable Resin/Polymer	High resolution	Volume shrinkage	Observation of cell growth	[21,22,23]
Acrylonitrile Butadiene Styrene (ABS)	Excellent surface finish, cheap material	Hydrophobicity	Pathogen detection, biological assays	[24,25,26]
Polyethylene Glycols (PEGs)	Relatively inexpensive, biocompatibility	Less cell adhesive, limited biodegradation	Microfluidic valves, microfluidic lifetime improvement	[15,27,28,29]
Polyhydroxyalkanoates (PHAs)	Biocompatibility, biodegradability	Hydrophilic, lacking sufficient moisture resistance	Barrier for vapor and oxygen	[30]
Gelatin Methacrylate (gel-MA)	Photopolymerizable, porous membrane	Weak mechanical properties, fast degradation	Vascular and valvular biology cell research	[31,32,33]
Polylactic Acid (PLA)Polyglycolic Acid (PGA)	Biodegradability	High degradation rate	Porous scaffolding, better adhesion	[15,34]
Styrene Ethylene Butylene Styrene (SEBS)	Biocompatibility, electrical surface properties, reduced drug absorption	Poor dimensional stability	Microdevices for electrokinetic applications, cell culture, complex networks	[35,36,37]

**Table 2:** Various liver-on-a-chip materials, sensors, and cell types.

Material	Sensor	Type of Cell	References
Polymethyl methacrylate (PMMA)	Electrochemical	Hepatocytes	[98]
Polycarbonate (PC)	Optical	Liver sinusoidal endothelial cells (LSECs)	[99]
Glass	pH	HepG2	[100]
Alginate	-	Hepatocytes, hepatic stellate cells, LSECs	[101]

**Table 4:** Disease models on the organ-on-a-chip.

Organ Modeled on Chip	Disease	References
Lung	Lung cancer, asthma, SARS-CoV-2	[160,161]
Liver	Hepatitis, liver fibrosis	[96,97]
Heart	Myocardial infraction, arrhythmias	[162]
Brain	Alzheimer’s disease	[108]
Intestine	Inflammatory bowel disease, colorectal cancer, GI bacterial infection, GI viral infection	[163]
BBB	Stroke, multiple sclerosis	[164]

## Data Availability

No new data were created or analyzed in this study. Data sharing is not applicable to this article.
